# Application of narrative in medical ethics

**DOI:** 10.18502/jmehm.v12i13.1642

**Published:** 2019-10-21

**Authors:** Saeideh Daryazadeh

**Affiliations:** *Ph.D. Candidate of Medical Education,* *Department of Medical Education, Medical Education Development Research Center (EDC), Isfahan University of Medical Sciences, Isfahan, Iran.*

## Introduction

**The Narrative Approach in Medicine**

 The use of narratives as narrative discourse is a tool used to express the experiences of individuals (1). In medical sciences, the use of the narrative approach has been emphasized by Rita Charon (2). Researchers who have used a narrative approach in medical education claim that practicing narrative writing can improve health care provision (3 - 6). In the health care system, narration is the usage of literature on patients' stories to facilitate clinical decision-making for physicians. Lately, the narrative has applied a healing potential as “narrative care”, a method that uses story to improve health care. This approach focuses on patients' stories (7). The narrative plays a very effective role in making difficult ethical decisions, and therefore it is important to keep considerations and consequences in mind (8).

With the growing use of narratives in medicine, its application as an educational tool in ethics has also increased (9). Narrative analysis is a way of examining ethical problems and the use of a narrative approach to ethical issues and values prevents ethical challenges (2). Fictional and factual narratives can be a prominent help to comprehension in medical ethics. Literary criticism can be used to analyze ethical texts, and this process enhances our understanding of different perspectives on ethical challenges (2). The issues of rights, errors, emotions, beliefs, and behaviors are related to ethical narratives (1).

**Narrative ethics**

Narrative ethics represents the postmodern critique of reason and emphasizes personal affective and creative interpretation. It originated in the domains of philosophy and theology. Narrative ethics is a component of effective ethics and deals with particular circumstances, supplying extreme consideration to the background that highlights the outcomes and concerns stated by a dilemma. Discursive representation is a narrative centering on a circumstance, and one that needs to be comprehended in a multidisciplinary way. These representations are stated in the form of narration, which offers an ethical approach (8). Narrative ethics is a term that contains a wide range of concepts presented as approaches; thus, narration can be a source of ethical theories or a method for studying ethics and can be a replacement for traditional methods. Hence, it is necessary to pay attention to the principles of narrative ethics. Firstly, ethical situations are unique and cannot be generalized to other issues, like rules. Secondly, narrative situations are decided based on the patient's status and his illness story (9). Usually, caregivers and doctors publish a case report. In reporting a patient's case, compliance with standards and principles is normally done, but the expression of the patient’s psychological features is neglected. While the narrative in the patient's file may seem to provide irrelevant information, it can also describe the patient's status to others. Therefore, the narrator presumes that in this way the ethical points will be better known (8). Narrative ethics is a current ethical view that has emerged lately and fosters effective interaction between patients and physicians; however, attention should be given to its application in the physician-patient communication (8).

**Narrative as a teaching method**

A review of several studies shows that medical students learn the most from clinical-role models, and the role of a clinical teacher as a model in the training of professional ethics is rather prominent (10, 11). However, there is a challenge that makes this impact undesirable. Since students are exposed to both favorable and unfavorable role model behaviors, they may be affected adversely under the circumstances. Also, the use of reflection and integration of humanities in medicine are other ways of teaching ethics to medical students (10, 12 - 14). Conducting discussions in small groups and role-playing or interacting with a patient as a teacher also plays an effective role in teaching medical ethics (10, 15). The above-mentioned methods, some of which are still not widely used, can be effective in medical education, but it is necessary to pay attention to cultural issues as an important point in education. Hence there is a need for a medical education system based on humanities (10, 16, 17).

Over the past few decades, narratives have been used as a method for teaching medical professional ethics. They provide ethical guidance through case examples, and as narratives of witness, address the review of professional ethical principles in medical practice (2, 18).

In medical ethics, narratives are used in the following three forms:

          1. Examples of cases that have broad application in Western medical ethics:

Since the advent of humanities in medicine in the 1970s, a link was formed between literature and medicine, and literature was employed to teach medical ethics. Literary narratives were used to analyze the ethical challenges in short stories in a designated framework. Hence, "stories as cases" are the principal method for teaching the principles of medical ethics (2).

          2. Moral guidance in all aspects of the lives of individuals:

From the perspective of Coles, complex literary texts such as novels are moral guides to making life better. Stories are not only used to solve moral challenges, but also to provide moral reflection on all of life's affairs, and as such, they are effective in medical ethics (2, 19).

          3. The “witness narratives” that are used to examine medical and ethical performance:

Analyses of ethical issues concerning patients or their relatives are important in terms of medical ethics. “Witness narratives” are related to issues such as independence, respect for patients’ rights, informed consent, and medical errors. Physicians and health care professionals share their experiences of such ethical issues and discuss the challenges. Therefore, the interpretation of "witness narratives" plays an important role in solving ethical problems (2, 20).

In summary, narratives can present ethical lessons, or create awareness and empathy through the expression of ethical issues and experiences. Also, narratives can help us by providing relevant information on "ethical decision-making". On the other hand, narratives can be a way of understanding the moral reasoning that is created through contemplation and exploration of individuals’ narratives (7). 

## Notes:

**Citation to this article:**

Daryazadeh S. Application of narrative in medical ethics. J Med Ethics Hist. 2019; 12: 13.
